# Internet of Plants: Machine Learning System for Bioimpedance-Based Plant Monitoring

**DOI:** 10.3390/s25247549

**Published:** 2025-12-12

**Authors:** Łukasz Matuszewski, Jakub Nikonowicz, Jakub Bonczyk, Mateusz Tychowski, Tomasz P. Wyka, Clément Duhart

**Affiliations:** 1 Faculty of Computing and Telecommunications, Poznań University of Technology, 60-965 Poznań, Poland; jakub.nikonowicz@put.poznan.pl (J.N.);; 2 Faculty of Biology, Adam Mickiewicz University, 61-614 Poznań, Poland; tomasz.wyka@amu.edu.pl; 3 IONIS Education Group, 75003 Paris, France

**Keywords:** Internet of Plants, plant-to-machine interface, biosensor, bioimpedance

## Abstract

Sensors in plant and crop monitoring play a key role in improving agricultural efficiency by enabling the collection of data on environmental conditions, soil moisture, temperature, sunlight, and nutrient levels. Traditionally, wide-scale wireless sensor networks (WSNs) gather this information in real-time, supporting the optimization of cultivation processes and plant management. Our paper proposes a novel “plant-to-machine” interface, which uses a plant-based biosensor as a primary data source. This model allows for direct monitoring of the plant’s physiological parameters and environmental interactions via Electrical Impedance Spectroscopy (EIS), aiming to reduce the reliance on extensive sensor networks. We present simple data-gathering hardware, a non-invasive single-wire connection, and a machine learning-based framework that supports the automatic analysis and interpretation of collected data. This approach seeks to simplify monitoring infrastructure and decrease the cost of digitizing crop monitoring. Preliminary results demonstrate the feasibility of the proposed model in monitoring plant responses to sunlight exposure.

## 1. Introduction

Integrating smart technologies into agriculture has led to the emergence of the Internet of Plants (IoP), an extension of the Internet of Things (IoT) paradigm that enhances crop monitoring and farm management. Traditionally, agricultural monitoring relies on WSNs to collect broad environmental data. While atmospheric parameters such as temperature and CO_2_ are diffusive and do not typically require high-density deployment, physiological stress factors—such as local water or light deficit—vary significantly between individual plants. Capturing this localized variability is essential for precision agriculture. Although WSN-based systems provide valuable information about plant growth conditions, large-scale deployment remains costly due to the high cost of electronic equipment and infrastructure maintenance. Despite advances in precision agriculture, the density of sensors remains sparse, with only one complex measuring station per 1000 m^2^ [1].

Recent developments in plant physiology and biotechnology suggest an alternative approach: utilizing plants themselves as biosensors [2]. Plants naturally respond to environmental stimuli through biochemical and electrical signaling mechanisms, including action and variation potentials. These intrinsic sensing capabilities can be used for plant-based monitoring systems, where plants act as biological sensors, interfacing with electronic components. Previous research has shown that plant electrical activity correlates with environmental factors such as light exposure, chemical presence, and mechanical stimulation [3,4,5]. However, most existing studies rely on active stimulation of plant tissues and real-time monitoring of rapid electrical changes, requiring continuous external intervention [6]. Our work builds upon these foundational principles but proposes a fundamentally different sensing modality and conceptualization of the IoP itself.

As introduced, e.g., by [7], this framework primarily treats IoP as an extension of the IoT, in which external devices collect and process plant-related data. In contrast, we redefine IoP as a paradigm shift from the conventional IoT. Rather than relying on external devices to monitor general field conditions, the IoP framework treats the plant itself as the primary, active data source. This shift allows for precision agriculture based on localized physiological feedback (e.g., specific stress responses) rather than broad environmental estimations, requiring a valid ‘plant-as-node’ interface which this study aims to establish. Rather than relying on transient electrical responses, we propose a sensing mechanism in which plants act as dynamic elements of an electrical circuit, specifically as bioimpedance components modulating circuit characteristics over time. This eliminates the need for external sensor nodes within WSNs, significantly simplifying the monitoring infrastructure. By integrating noninvasive single-wire connections, low-cost hardware, and machine learning-based data processing, our system enables continuous monitoring of plant responses to environmental stimuli, supports near-real-time intervention and control, and ensures optimal growth conditions. This positions plants as active, circuit-integrated components within the IoT ecosystem, combining plant-based sensing with AI-driven analysis. Consequently, our approach supports the practical implementation and scalable adoption of the IoP paradigm within smart farming and precision agriculture.

This paper introduces the design and validation of the novel plant-to-machine interface that enables this redefined IoP network. Our approach aligns with current trends in AI-driven agriculture [8], where the growing adoption of edge computing and System-on-Chip (SoC) architectures facilitates near-real-time, decentralized data processing [9]. By integrating our bioimpedance sensor with AI-driven edge processing, we create a practical path for incorporating neural networks into embedded hardware for localized decision-making. The feasibility of this entire model is validated through experimental results on light exposure, confirming its potential as a simplified and cost-effective data acquisition method for smart agriculture applications.

## 2. IoP Architecture

**Figure 1:**
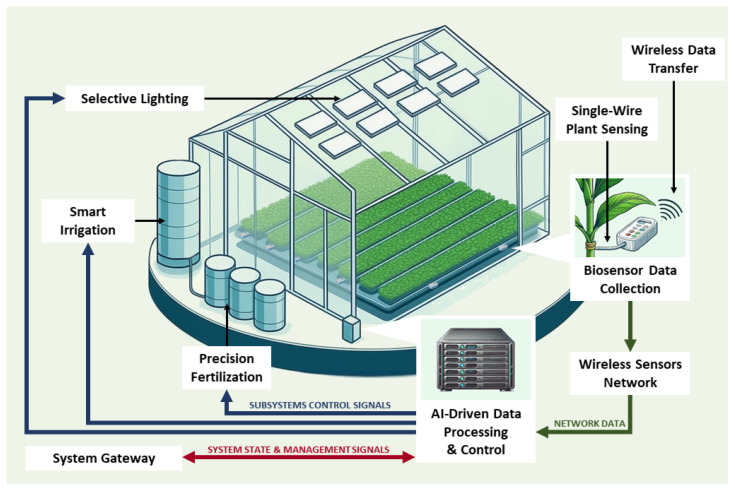
A high-level vision of the proposed IoP architecture. The system integrates a plant-based biosensor into a feedback loop with AI-driven data processing and environmental control subsystems, illustrating the flow of data and control signals.

Realization of the presented IoP vision, in which the plant becomes an active component of the network, requires a hybrid architecture that integrates plant-based biosensing with data processing and environmental control modules. To translate this concept into a functional system, we designed a scalable architecture consisting of the following key elements (Figure 1):

1. Plant-based biosensor—The plant functions as a living biosensor whose physiological responses to environmental stimuli cause measurable changes in its bioimpedance. This electrical impedance spectrum (EIS) is periodically measured to infer the plant’s condition and surrounding microclimate.
2. Non-invasive single-wire interface—A single-wire electrode establishes a direct, non-damaging electrical connection for impedance measurements, ensuring signal integrity during long-term monitoring.
3. Embedded measurement unit—A low-power microcontroller (e.g., ESP32, STM32, Raspberry Pi Pico) performs three core functions:
  - Frequency sweep generation—The unit generates a controlled frequency range to excite the plant-circuit system.
  - Electrical response acquisition—It records the system’s response to form a characteristic plant’s EIS.
  - Wireless data transmission—It transmits the captured spectrum to the AI processing stage for analysis.
4. Data processing and AI-based classification—This stage analyzes the bioimpedance data using a neural network (NN) in one of two deployment models:
  - Centralized processing—Data is transmitted to a server where a continuously retrained NN (*online learning*) classifies plant conditions, adapting to long-term physiological changes.
  - Edge processing—Alternatively, the NN model runs locally on the microcontroller or an edge AI chip, enabling adaptive responses with minimal latency and no cloud dependency.
5. Environmental control system—Triggered by the AI classification results, this system uses automated actuators to optimize growth conditions. It adjusts selective lighting, smart irrigation, and microdosed fertilization within an adaptive feedback loop.
6. Remote monitoring and management—A cloud-based dashboard or mobile application provides users with near-real-time data on plant health and environmental conditions. It allows for remote system configuration and delivers alerts based on AI-driven analysis.

### 2.1. Plant as a Sensor

The core of our system relies on EIS to interpret the plant’s physiological state. While other non-invasive techniques like thermal imaging or chlorophyll fluorescence provide valuable spatial data on plant health, they often require complex and costly equipment. EIS offers a compelling alternative focused on simplicity and cost-effectiveness. By utilizing a single-wire, low-cost interface, bioimpedance measurements provide direct insight into the plant’s bulk physiological state. To understand the origin of this signal, it is essential to consider the electrical properties of plants.

To a first approximation, the electrical properties of plants arise from their rich chemical composition, characterized by numerous charged elemental and compound molecules distributed nonuniformly throughout the plant body. At the level of individual living cells, charge gradients are established and maintained across the plasma membranes and organellar membranes (e.g., tonoplast, plastid envelopes, and thylakoids) due to the activity of diverse ion channels. These gradients are essential for driving cellular transport and metabolism.

At the tissue scale, transmembrane potentials translate into electrical potentials between the symplast and the generally more negative apoplast. Intercellular, tissue-level, and organ-level communication includes waves of electrical depolarization and repolarization of plasma membranes, which typically propagate in an orderly manner along specific routes such as the phloem [10]. Long-distance ion transport in specialized vascular tissues can generate electrical potentials, such as streaming potentials associated with xylem sap flow [11,12]. An electrical potential gradient develops along the plant axis at the whole plant scale [11]. These whole plant potentials exhibit seasonal and diurnal variability and may be inversely related to the transpiration rate [12].

Many of these electrical processes are actively generated and utilized by the plant. Regulated ion channels and ion transporters facilitate the uptake, transport, and extrusion of ions [13], and transmit signals between cells over long distances, such as from leaf to leaf or root to shoot [10]. Electrical signaling between organs is particularly important in coordinating systemic responses of plants to environmental stresses and stimuli [4,5].

Plant structure plays a key role in shaping electrical phenomena by providing low- and high-resistance pathways for current flow. Sieve elements, phloem parenchyma cells, and vascular bundle sheaths are preferentially involved in long-distance electrical signal transmission. Tracheary elements within the xylem, as well as the sieve tubes, support the long-distance movement of ions and their associated charges [10,11]. In contrast, high electric resistances occur across structural barriers such as the endodermis, exodermis, and other secondary-wall rich tissues, as well as air spaces.

Electrical impedance measures an object’s ability to resist the flow of electrical currents. In whole plants, bioimpedance integrates the effects of ionic content and distribution, intercellular connections (plasmodesmata), as well as anatomical connections (vascular bundles) and barriers, and ongoing solute flows within the plant [14]. Depending on the measurement timescale, the bioimpedance variation can capture both baseline physiological processes and environmental influences. Due to the complexity of plant structure and internal electrical dynamics, the mechanistic link between physiological processes and the bulk impedance signal remains poorly understood. Nonetheless, empirical studies have successfully associated bioimpedance shifts with dehydration [15,16] and salinity stress [17], nutrient deficits [18], and other stress conditions [14]. For example, diurnal changes in tree water content have been shown to negatively correlate with electrical resistance, a component of bioimpedance, between the tree and the soil [12].

Photosynthesis and associated gas exchange may be a major source of temporal variation in electrical properties of the entire plant. Stomatal opening allows transpiration, which drives the flow of xylem sap and reduces the water content of the tissue, often accompanied by the formation of discontinuities in the xylem water column (so-called emboli), potentially increasing the impedance. Conversely, the availability of assimilates activates phloem transport of sugars along with various charged molecules. Light can also activate various transporters and channel proteins, including aquaporins [19]. Because the effects of these processes on bulk plant impedance are still not well understood, the development of IoP tools for monitoring overall plant stress may benefit from studying impedance in plants subjected to varying levels of factors that control photosynthesis, such as light intensity or moisture stress.

### 2.2. Plant-to-Machine Interface

Frequency-swept capacitive touch sensors are a technology that enables stimulus detection and the analysis of more subtle interactions. This technique generates a variable-frequency signal and monitors the system response to each frequency. The fundamental assumption of this approach is that different objects can influence the measurement system in distinct ways, depending on the frequency of the test signal. Such a sensor can use a single electrode connected to the object intended as the stimulus detector instead of employing a more complex grid of electrodes. An example of a system implementing the sensor mentioned above is presented in the article [20]. The system uses a frequency generator to synthesize sinusoidal signals from 1 kHz to 3.5 MHz with a step of 17.5 kHz. The signal is filtered to minimize environmental impact and unwanted higher-frequency components. Subsequently, it is amplified and stimulates the object connected via the electrode. A coil with high inductance is used to enable the sensor to detect small changes in the object’s capacitance. After filtering and envelope detection, the signal is fed into an analog-to-digital converter in the microcontroller.

Frequency-swept capacitive sensors have not yet found commercial applications. This technique requires complex data processing to recognize events. Machine learning algorithms require significant computational power and are most commonly used for event recognition.

**Figure 2:**
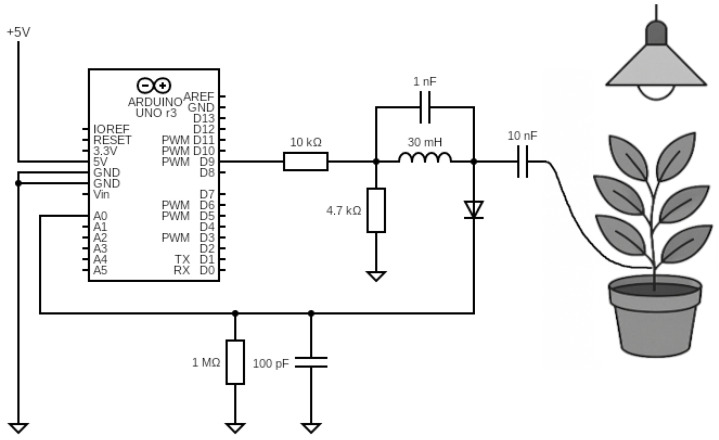
Plant-to-machine interface circuit adapted for the experimental setup, utilizing an Arduino development board for data acquisition and transmission. Detailed component values are provided in Section 2.2.

The present work implemented a much simplified sensor version utilizing a 32-bit system-on-chip (SoC) evaluation board—specifically the Arduino Nano Connect RP2040—and a few discrete passive electronic components (Figure 2). While commercial solutions for physiological monitoring, such as sap flow sensors, dendrometers, or advanced stomatal conductance systems, typically incur costs ranging from $500 to over $2000 per node, the proposed measurement unit can be assembled for less than $40. By reducing the financial barrier by nearly two orders of magnitude, this cost efficiency serves as a key enabler for the high-density deployment required by the Internet of Plants paradigm. Although the measurement capabilities of the proposed prototype do not yet equal the precision of high-end commercial equivalents, this economic accessibility positions it as a scalable developmental base, enabling the expansion of these capabilities with additional data modalities (see Section 4).

The working principle of the sensor is identical to the frequency-swept stimulus sensor described above. The microcontroller produces a square wave signal to simplify the design instead of generating sinusoidal signals. Many harmonic frequencies characterize this signal, instead of measuring the signal response directly before the LC filter coil is connected to the tested object. This system measures the response attached to the object. In this case, the object is a plant connected to the electronic circuit with a single wire, which forms an impedance system with a characteristic similar to a band-pass filter [21].

**Figure 3:**
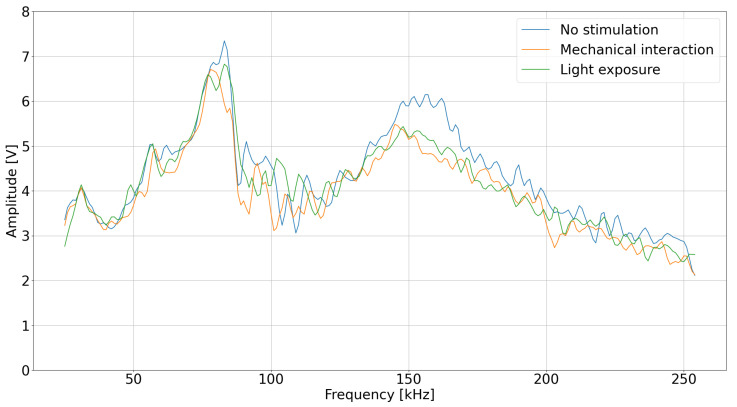
Bandpass filter stimulus reaction.

The microcontroller generates a variable frequency square wave signal that passes through a series resistor (10 kΩ) and a parallel resonant tank (*L* = 30 mH, *C* = 1 nF, $R_{damp}$ = 4.7 kΩ) before coupling to the plant via a 10 nF capacitor. This topology forms a band-pass filter with a center frequency of $f_{0}\approx$ 125 kHz, and 3 dB bandwidth of ≈150 kHz, where the plant acts as a complex load modulating the circuit’s impedance. The signal then passes through an envelope detector (series diode, parallel *R* = 1 MΩ, *C* = 100 pF) with a cutoff frequency of ≈1.6 kHz, is sampled, and stored in memory. By reading the amplitude value on the system output for each frequency that excites it, a signal is formed that resembles the characteristic of a band-pass filter. Interaction with the object causes a change in the impedance of the LC-object system, resulting in a change in the amplitude–frequency response. The system captures changes in the plant’s impedance. The data is transferred to the microcontroller and then to the computer for further processing. During broadband measurement tests, it was possible to examine the necessary frequency band from a starting frequency of 20 kHz to an end frequency of 250 kHz with a step of 1 kHz (Figure 3). The frequency range was selected to maximize the observed plant response and ensure noticeable changes in the system’s impedance within this band, as variations outside this range appeared less significant during preliminary testing.

### 2.3. Connectivity and Data Processing

The plant-to-machine interface collects frequency response data using the single-wire interface, wherein each frequency sweep consists of multiple measurement points. The generation and processing of these frequency points depend heavily on the hardware responsible for implementing the measurement units. In this context, a microcontroller enables configurable frequency sweep generation, offering flexibility in the bandwidth utilized for transmitting frequency points. Subsequently, a sample of data collected from a plant forms a two-dimensional matrix, in which the rows correspond to a time-series sweep and each column represents a particular frequency bin. Importantly, each sample must be appropriately labeled according to the current plant condition to ensure the data can be used effectively for model training.

**Figure 4:**
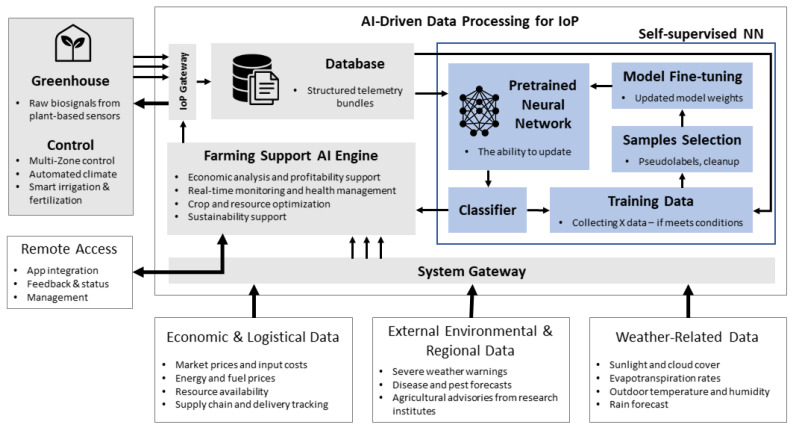
Information processing in AI-augmented crop monitoring via IoP network integration.

Furthermore, this arrangement allows for the continuous transmission of raw biosignal data to a centralized IoP gateway for data aggregation. The aggregated data are then periodically stored in a dedicated database, ensuring long-term availability for subsequent analysis (Figure 4).

Once the data are collected and securely stored, they can be processed by self-supervised neural networks. Deep learning techniques are applied to analyze plant data and classify various plant conditions in this stage. Neural networks are particularly effective in capturing both the temporal and spatial patterns inherent in biosignal data [22,23]. However, it must be emphasized that machine learning algorithms, particularly deep learning models, require substantial amounts of labeled data to achieve high performance. Within this framework, the classifier component is tasked with inferring classification data, which is subsequently transferred to the AI agriculture support engine. If the inferred data satisfy predefined conditions, it is utilized to fine-tune the model, thereby updating its internal weights. Consequently, the IoP system, through the deployment of conventional sensors, can generate large datasets, enabling continuous learning and ongoing model refinement.

Moreover, the AI engine for agricultural support can analyze the neural network output and the data collected at the system gateway. Based on this analysis, it can provide critical information on crop profitability, crop optimization, and sustainability support. In practice, this refers to the function of an AI-based system that analyzes environmental and production data to support decision-making and sustainable crop management. This includes recommending strategies that increase resource efficiency, minimize environmental impact, and ensure long-term sustainability of agricultural production.

Finally, the data provided by the system gateway encompasses not only biosignal information but also contextual data related to economic, logistic, environmental, and regional factors. This comprehensive dataset ensures a holistic understanding of the greenhouse operation and its broader impact.

## 3. Experimental Setup and Preliminary Results

The experimental setup was designed to validate the fundamental unit of the IoP architecture: the single-plant node. By demonstrating that a low-cost hardware interface coupled with an AI processing pipeline can successfully digitize and interpret the physiological state of a single plant, we establish the feasibility of scaling this architecture to a network of thousands of such nodes.

The experiment was conducted to check whether detecting when a plant is illuminated and for how long using a simple system is possible. Conducted under controlled conditions in a grow box with stable temperature and humidity, the test involved periodic light exposure using an artificial sunlight-mimicking lamp. Data collection covered both dark and light-exposed phases, enabling the system to learn recognition of these states and their temporal patterns through specialized machine learning algorithms. Through multiple iterations with varying exposure durations and algorithm parameter adjustments, we evaluated the system’s light-state classification accuracy.

### 3.1. Tent Environment

**Figure 5:**
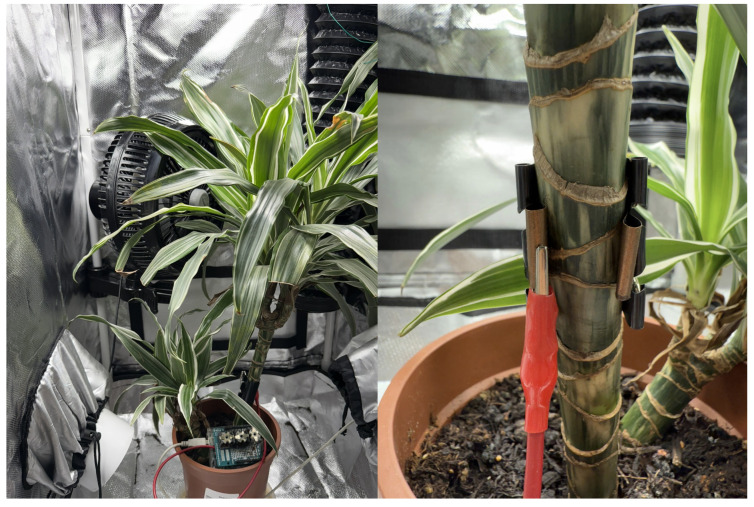
Experiment equipment (**left**), with non-invasive one-wire connection (**right**).

The study employed distinct individual specimens from two morphologically diverse species: *Zamioculcas zamiifolia* (succulent stem) and *Dracaena deremensis* (woody stem). This selection was designed to validate the versatility of the non-invasive strap interface across different plant structures. The primary focus of this work is to confirm the hardware’s signal acquisition sensitivity and the machine learning pipeline’s adaptability to different biological baselines. Plants from a local market were placed in a grow box, a special tent (50 × 50 × 140 cm) that controls environmental conditions such as humidity, temperature, and light (Figure 5). Environmental conditions inside the tent were regulated using a precision thermostat integrated with an air-conditioning system and a water-mist hygrometer for humidity control. Throughout the experiment, the temperature inside the tent was kept at 23 °C, and the relative humidity was 30%.

The exposure time was systematically varied according to the design of the experiment (in the range from 1 to 4 h). The plant was integrated with the previously described stimulus–response detection system via a non-invasive single-wire interface via a non-invasive single-wire interface. As shown in the close-up in Figure 5, this interface consists of a flexible conductive strap wrapped securely over the plant stalk. This design ensures a consistent capacitive coupling surface area while accommodating the natural curvature of the stem without damaging the epidermis. However, the interface can be attached to any plant part [21], enabling direct measurement of electrical signals within the plant tissue. Moreover, this interface does not affect the plant’s natural processes and growth patterns.

A microcontroller mounted on the PCB controlled the measurement frequency sweep and transmitted data to the computer. The computer processed the acquired information and performed a machine-learning process to analyze the collected data. This setup enabled effective identification of the plant’s exposure state—whether in light or darkness—based on the electrical response.

**Figure 6:**
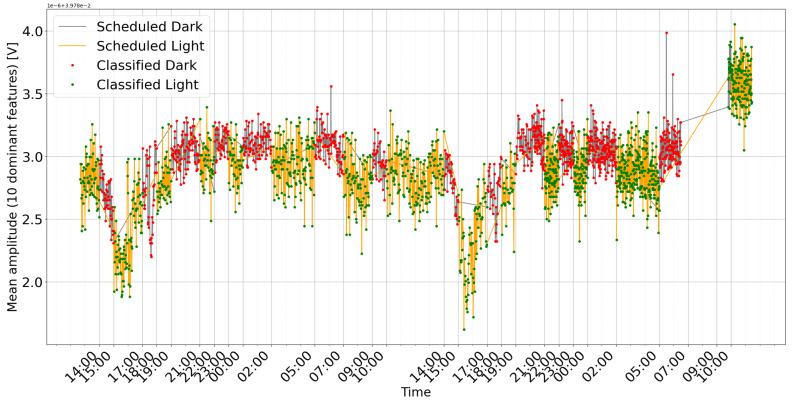
Mean amplitude of the 10 dominant frequency components in the *Dracaena* plant’s response time series, color-coded by scheduled light/dark periods and annotated with the corresponding classification mode (light or dark).

The lights in the grow box were turned on and off at variable time intervals to create alternating phases of light and darkness. The model learns to identify when light or darkness occurs, and the variability in phase duration prevents it from classifying changes based solely on a fixed time pattern. Figure 6 shows an example of detection during cyclic changes with a variable time period. During the experiment, measurements were taken every minute. Each measurement consisted of 100 frequency sweeps.

The data were subjected to preprocessing before being fed into the machine learning model. This stage consisted of normalization using variance and standard deviation to improve the model’s performance. The normalized dataset was then used to train a simple recurrent neural network architecture with a long short-term memory (LSTM) layer. After training and evaluating the model’s performance, it was directly fed data from the plant. The classification results were saved for further analysis. The model could recognize changes in lighting conditions, although with a delay of several minutes. This could be related to the plant’s response time to external stimuli.

During the experiment, concerns were raised that the model might have learned to associate light exposure with fixed time intervals, rather than detecting real physiological responses. To address this issue, an additional experiment was conducted in which the light exposure pattern was deliberately altered after training. The model, connected directly to the sensor stream, was then evaluated under conditions where lights were turned on during expected dark periods.

### 3.2. Data Collection and Organization

The plant-to-machine interface is designed to capture the plant’s electrical frequency response by measuring 230 distinct frequencies per sweep. Each sweep is transmitted via a UDP packet over the wireless network to the collector. With each frequency point encoded in 2 bytes, a single sweep occupies roughly 460 bytes. This configuration was selected to balance the need for high-resolution frequency data with the constraints of the UDP packet payload size and sweep duration.

Microcontroller samples frequencies at a rate of 1000 Hz. It excites the object with a specified frequency, waits for 1 millisecond, and reads the amplitude value. Data acquisition is performed in 1-min intervals. In each interval, the system activates a ‘burst’ recording mode, capturing 100 consecutive frequency sweeps over a duration of approximately 23 s. Since each sweep covers 230 distinct frequency points, this results in a data package of dimensions 100×230 (sweeps × frequencies) per minute. This burst approach ensures high temporal resolution of rapid physiological changes while maintaining a manageable data transmission schedule. A transmission rate of roughly 46 kbps is required.

The dataset is stored in a structured format with three primary columns:

- times: Specifies the number of measurements per frequency, ensuring temporal alignment across sweeps.
- data: Contains a 100 × 1 × 230 array, representing 100 sweeps of 230 frequency measurements each. This multidimensional format supports both temporal and spectral analysis.
- label: Indicates the environmental condition under which the data was recorded, typically “dark” or “light”. This binary labeling is central to the experiment’s aim of differentiating plant responses under varying lighting conditions.

The labeling follows a repeating hourly schedule, alternating the light conditions at irregular intervals ranging from one to three hours. This controlled environment enables the study to isolate the effects of light exposure on plant responses.

Before analysis, the dataset undergoes a filtering process based on the experimental protocol. Specifically, the first 10 min of data following a transition in light conditions (either turning on or off) are removed. This filtering is justified by the observation that transient effects immediately after a light change can introduce noise and variability, which might confuse the model’s ability to classify the plant’s steady-state response accurately.

To ensure that all features contribute equally to the model training process, the remaining data is standardized using the StandardScaler function from the sklearn library. Standardization normalizes the data to have a mean of 0 and a standard deviation of 1, which is crucial for the stability and performance of the subsequent neural network training.

### 3.3. Model Architecture and Training

The experiment employs a Long Short-Term Memory (LSTM) based neural network to classify the plant’s state based on its frequency response. The model is structured with LSTM, Attention, and Dense layers. The first layer processes the input sequence; a custom Attention layer is then applied to compute a weighted sum of the LSTM output across all timesteps. Subsequently, a Dense layer with 16 neurons performs non-linear feature transformation, followed by a final Dense layer with a single Sigmoid-activated neuron that outputs a probability score (0 to 1) suitable for the binary classification task. The complete neural network includes:

- An LSTM layer with 16 units and return_sequences = True which ensures that the output sequences are 16-dimensional vectors, preserving information for the input of the Attention layer.
- An Attention layer, which is a custom class implementing a form of additive attention to calculate weights for the Dense layer.
- A Dense layer with 16 units, ReLu activation function, and l2 regularizer with a factor of 1 × 10^−3^ to prevent overfitting.
- A Desnse layer with 1 unit and Sigmoid function that performs binary classification.

To further mitigate the risk of overfitting, dropout rates of 0.5 are applied in various layers. Additionally, two Keras callbacks—EarlyStopping and ReduceLROnPlateau—are integrated into the training process. These callbacks adjust the learning rate dynamically and stop training when improvements stagnate, ensuring the model remains generalizable to unseen data. The model training process adhered to a strict chronological split to respect the time-series nature of the data. The dataset was divided as follows: the first 60% of the time-series data was allocated for training, the subsequent 20% for validation, and the final 20% for testing. The network was trained using the Adam optimizer with an initial learning rate of 1 × 10^−3^ and a batch size of 32. Training was capped at 100 epochs, with an EarlyStopping callback triggered if validation loss failed to improve for 10 consecutive epochs. Furthermore, maintaining a balanced class distribution across the training, validation, and testing sets is critical to prevent the model from developing a bias towards the majority class. Consistent with the binary classification task, a binary cross-entropy loss function was employed. Following training on the initial dataset (derived from the first plant), the model achieved high validation accuracy (98%). However, when applied directly to data collected from a different plant specimen without retraining, the model’s accuracy dropped significantly to 57%. To address this performance degradation, a fine-tuning strategy was implemented. This involved applying the same chronological data partitioning to the new dataset, utilizing the Adam optimizer with a reduced learning rate of 5 × 10^−4^, and employing the previously defined callbacks (EarlyStopping, ReduceLROnPlateau) with adjusted parameters. After fine-tuning on the second plant’s dataset (approximately 4 GB), the model achieved a final test accuracy of 92%.

**Figure 7:**
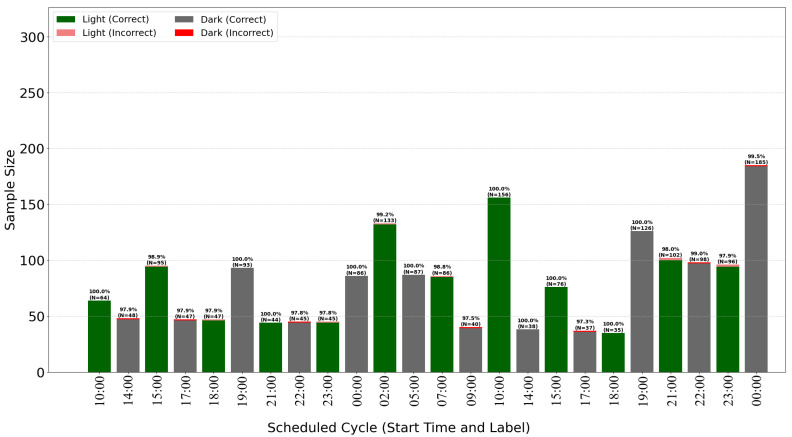
Time of exposure to dark/light with models predicion for *Dracaena*.

**Figure 8:**
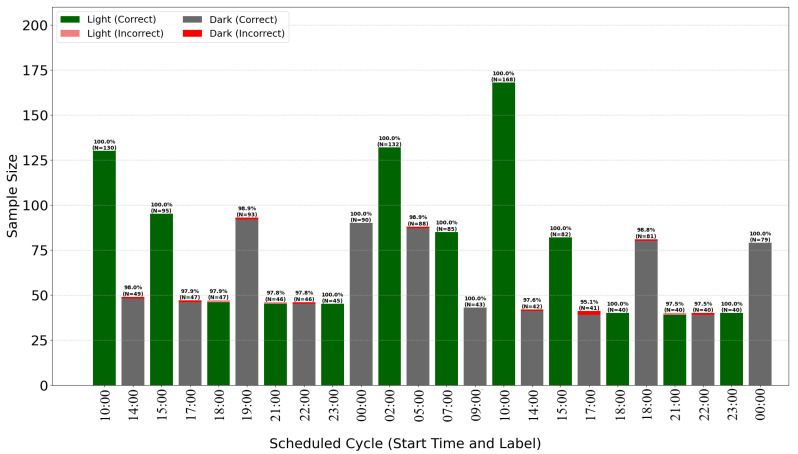
Time of exposure to dark/light with models predicion for *Zamioculcas*.

The breakdown of classification accuracy per cycle (Figure 7 and Figure 8) demonstrates the model’s high overall effectiveness, with only sporadic errors. Analysis of misclassifications (red and pink bars) suggests they occur primarily during transitional periods or specific cycles, providing insights for further algorithm optimization.

### 3.4. Feature Importance Analysis and Visualization

To understand the model’s decision-making process, integrated gradients are used to assess the impact of each input feature on the output predictions. This method gradually transitions the input from a baseline state to its actual value while computing gradients at each step. The resulting gradients are averaged to produce an importance score for each feature at each time step. Summing these scores over time yields a single importance value per feature, allowing the identification of the most influential factors in the model predictions. This analysis validates the model’s performance and provides insights into the underlying biological processes.

**Figure 9:**
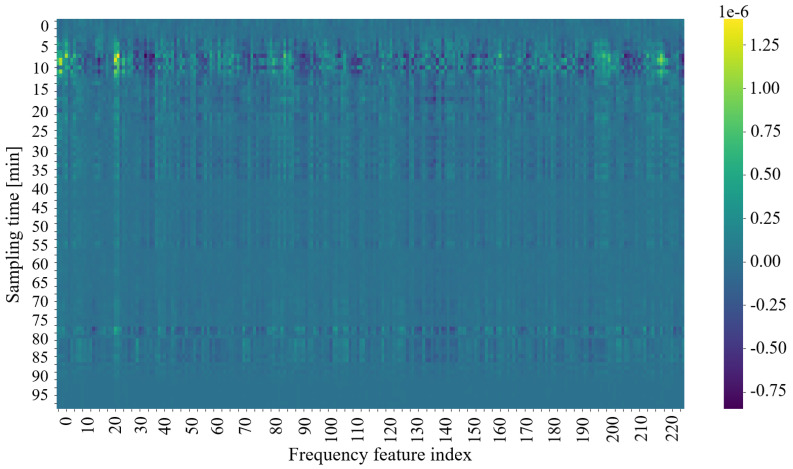
Feature’s weight in relation to model classification.

**Figure 10:**
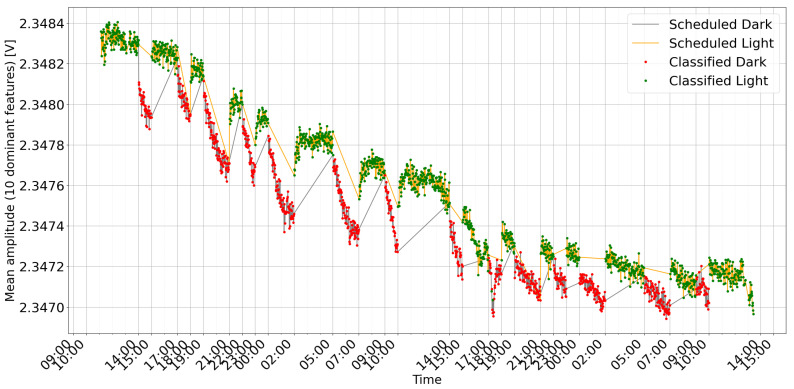
Mean amplitude of the 10 dominant frequency components in the *Zamioculcas* plant’s response time series, color-coded by scheduled light/dark periods and annotated with the corresponding classification mode (light or dark).

To further elucidate the model’s behavior, two visualization approaches are employed:

- Heatmap Representation: A heatmap is generated from the gradient weights of a single input sample (see Figure 9). Organized as a 100 × 230 matrix, the heatmap visually represents the importance of the characteristics.
  - The 100 rows represent time steps, highlighting how the importance of the feature evolves.
  - The 230 columns correspond to individual frequency measurements, indicating their respective contributions to the model’s decision.
- Feature Trends: Ten key features, determined by their high importance scores, are averaged and plotted over time alongside the model’s classifications and the actual labels (see Figure 6 and Figure 10). This graph helps to correlate specific frequency responses with the light/dark conditions.

The values in the heat map represent the calculated gradients; higher values denote a stronger influence on the classification outcome. This visualization supports the conclusion that a subset of the 230 frequencies may be sufficient to determine the plant’s exposure to light, thus guiding future experimental design and potential optimizations in sensor configuration. Analysis of the heatmap suggests that frequencies within the 0–30 kHz and 190–220 kHz ranges are particularly influential, potentially indicating specific physiological responses correlating strongest with light changes.

The experimental design is driven by the need to accurately capture and interpret the plant’s response to environmental changes, particularly light exposure. By using a high-resolution frequency sweep and structuring the data into manageable samples, the study ensures that transient effects are minimized and that steady-state responses are reliably recorded. The use of an LSTM is particularly suited for this type of data, enabling the extraction of both local and global patterns. Furthermore, the integrated gradient analysis and visualization techniques validate the model’s predictive power and provide biological insights, which are essential for optimizing both the sensing technology and the data analysis pipeline.

Detailed data acquisition, preprocessing, and application of machine learning methods allow the study of plant responses under varying environmental conditions. This comprehensive approach justifies the experimental choices and sets the groundwork for future enhancements to hardware and software components.

## 4. Discussion and Conclusions

The observed changes in plant bioimpedance features in response to light shifts (Figure 6 and Figure 10) support the hypothesis that impedance is linked to light-driven metabolic activity. It is important to note that light exposure simultaneously drives transpiration, stomatal opening, and local temperature variations. Therefore, the impedance signal likely represents a composite of these interconnected physiological processes rather than photosynthesis in isolation. The time series plots clearly correlate with the scheduled light/dark cycles, visually confirming the system’s ability to track the plant’s response. The observed delay in signal change after light switching, along with the noticeable baseline drift (especially in Figure 10), reflects the natural dynamics of the plant’s physiological processes and the complexity of the bioimpedance signal. Logical further steps should include calibrating bioimpedance signals against direct metrics of photosynthetic activity and investigating their relationship to environmental stress levels. Due to its reliance on a wide frequency range, the method we propose has the potential to detect declines in photosynthesis and disentangle the impact of specific stress factors, such as drought, sub- and supra-optimal temperature, and excess or deficit of irradiance.

Given the ubiquity of systemic electrical signaling in plants [24,25,26], an additional direction for bioimpedance monitoring not explicitly considered in this study would be to tune into specific characteristics associated with stress signals generated by the plant, such as changes in membrane potential in response to mechanical damage, water deficit, temperature extremes, or pathogen infections. For example, action and slow wave potentials can propagate along the conducting tissue and influence local and distant physiological responses, such as stomatal closure or activation of defense pathways. Bioimpedance analysis could therefore be used to detect characteristic patterns of electrical conductivity corresponding to specific stressors, thereby opening the possibility of using this method in plant health monitoring or near-real-time detection of plant responses to environmental factors [27].

In the long term, growth and development will modify the plant’s bulk electrical impedance. For example, accumulating secondary tissues and lignifying cell walls are expected to increase the impedance, while tissue and organ turnover will also introduce changes. These dynamic physiological processes necessitate periodic recalibration of baseline signals and retraining of the model to maintain classification accuracy. Therefore, long-term monitoring systems must incorporate mechanisms for adaptive learning and baseline drift correction to remain responsive to the evolving bioelectrical properties of the plant. One possible solution is an adaptive learning system that uses labeled data from a previously trained model to retrain a new one, potentially correcting for signal drift caused by physiological changes. However, such a system must account for the risk of amplifying classification errors, especially if the labels provided by the original model are inaccurate. Thus, the learning process must be carefully monitored to ensure the model’s continued accuracy.

This sensor system could be deployed in a variety of agricultural settings, including greenhouse crops, outdoor nurseries, and plantations. Key factors that impact its usability include ease of installation and the permanence of the probe. In this respect, tree trunks appear to be promising sites for probe placement, with conifers offering the added advantage of low annual biomass turnover. However, it is important to acknowledge the limitations of this study. The presented experiment serves as a proof-of-concept, conducted under highly controlled laboratory conditions on two ornamental species (*Zamioculcas zamiifolia*, *Dracaena deremensis*). While these results demonstrate the feasibility of the proposed method, further validation on key agricultural crops, such as corn or wheat, is essential. Moreover, future research must address the challenge of applying this technique in real-world field conditions, where a multitude of interacting environmental factors (e.g., wind, rain, pests) can influence plant bioimpedance. Disentangling these complex signals to isolate specific stress responses remains a significant but critical challenge for the practical, large-scale deployment of this technology.

In conclusion, this work introduces and validates a novel plant-to-machine interface based on EIS. By redefining the plant as an active, integrated component within an IoP network, we demonstrate a feasible path toward low-cost, scalable crop monitoring. While significant challenges related to real-world deployment and signal complexity remain, this proof-of-concept establishes a foundational methodology for a new generation of smart agriculture systems that listen directly to the plants themselves.

## Data Availability

The original contributions presented in this study are included in the article. Further inquiries can be directed to the corresponding author.
